# Physicochemical and Microbiological Assessment of an Experimental Composite Doped with Triclosan-Loaded Halloysite Nanotubes

**DOI:** 10.3390/ma11071080

**Published:** 2018-06-25

**Authors:** Diana A. Cunha, Nara S. Rodrigues, Lidiane C. Souza, Diego Lomonaco, Flávia P. Rodrigues, Felipe W. Degrazia, Fabrício M. Collares, Salvatore Sauro, Vicente P. A. Saboia

**Affiliations:** 1 Post-Graduate Programme in Dentistry, Federal University of Ceará, Rua Monsenhor Furtado S/N, Rodolfo Teófilo, Fortaleza 60430-355, Ceará, Brazil; araujo.diana@gmail.com (D.A.C.); nara.sousa.rodrigues@gmail.com (N.S.R.); lidiane_costa26@hotmail.com (L.C.S.); lomonaco@ufc.br (D.L.); flapiro@gmail.com (F.P.R.); vpsaboia@yahoo.com (V.P.A.S.); 2 Department of Organic and Inorganic Chemistry, Federal University of Ceará, Fortaleza 60440-900, Ceará, Brazil; 3 School of Dentistry, Paulista University—UNIP, R. Dr. Bacelar 1212, Vila Clementino, São Paulo 04026-002, SP, Brazil; 4 Laboratório de Materiais Dentários, Faculdade de Odontologia, Universidade Federal do Rio Grande do Sul, Rua Ramiro Barcelos, 2492, Rio Branco, Porto Alegre 90035-003, Rio Grande do Sul, Brazil; fdegrazia@hotmail.com (F.W.D.); fabricio.collares@ufrgs.br (F.M.C.); 5 Departamento de Odontología, Facultad de Ciencias de la Salud, Universidad CEU-Cardenal Herrera, C/Del Pozos/n, Alfara del Patriarca, 46115 Valencia, Spain; 6 Tissue Engineering and Biophotonics Research Division King’s College London Dental Institute (KCLDI), London SE1 9RT, UK; 7 Department of Restorative Dentistry, School of Dentistry, of Ceará, Fortaleza 60430-355, Ceará, Brazil

**Keywords:** mechanical properties, nanotubes, resin composite, *Streptococcus mutans*, triclosan

## Abstract

This study is aimed at evaluating the effects of triclosan-encapsulated halloysite nanotubes (HNT/TCN) on the physicochemical and microbiological properties of an experimental dental composite. A resin composite doped with HNT/TCN (8% *w*/*w*), a control resin composite without nanotubes (HNT/TCN-0%) and a commercial nanofilled resin (CN) were assessed for degree of conversion (DC), flexural strength (FS), flexural modulus (FM), polymerization stress (PS), dynamic thermomechanical (DMA) and thermogravimetric analysis (TGA). The antibacterial properties (M) were also evaluated using a 5-day biofilm assay (CFU/mL). Data was submitted to one-way ANOVA and Tukey tests*.* There was no significant statistical difference in DC, FM and RU between the tested composites (*p* > 0.05). The FS and CN values attained with the HNT/TCN composite were higher (*p* < 0.05) than those obtained with the HNT/TCN-0%. The DMA analysis showed significant differences in the TAN δ (*p =* 0.006) and Tg (*p =* 0) between the groups. TGA curves showed significant differences between the groups in terms of degradation (*p =* 0.046) and weight loss (*p =* 0.317). The addition of HNT/TCN induced higher PS, although no significant antimicrobial effect was observed (*p =* 0.977) between the groups for CFUs and (*p =* 0.557) dry weight. The incorporation of HNT/TCN showed improvements in physicochemical and mechanical properties of resin composites. Such material may represent an alternative choice for therapeutic restorative treatments, although no significance was found in terms of antibacterial properties. However, it is possible that current antibacterial tests, as the one used in this laboratory study, may not be totally appropriate for the evaluation of resin composites, unless accompanied with aging protocols (e.g., thermocycling and load cycling) that allow the release of therapeutic agents incorporated in such materials.

## 1. Introduction

Resin dental composites (RDCs) have been widely modified in the last few decades. Indeed, in order to increase their clinical performance, especially in terms of wear resistance and lower polymerization shrinkage, modern RDCs are formulated with a high amount of glass/ceramic fillers (60–80 wt.%) [1]. Moreover, inorganic nanoparticles and nanofibers have also been incorporated within the composition of RDCs to advance their mechanical and esthetic properties [2], as well as their biological and bioactive properties [3].

Nowadays, the formation of secondary caries remains one of the main reasons for the replacement of resin composite restorations [4,5,6]. Carious lesions along the margins of our restorations present an important causal relationship with the accumulation of a cariogenic biofilm; this is probably facilitated by gaps formed at the tooth-restoration interface, as well as by excessive roughness of resin composite [7]. *Streptococcus mutans* is one of the main species of a cariogenic biofilm responsible for secondary caries [8], hence, restorative materials with bioactive and antimicrobial properties are needed in order to improve the clinical outcome in daily practice [9].

Triclosan (TCN) is a well-known antibacterial agent used in a wide range of products such as toothpastes and mouthwashes. Several studies demonstrated the efficiency of such therapeutic substances against gram-positive microorganisms, (e.g., *Streptococcus mutans* [10,11], *Staphylococcus aureus* [12], *Lactobacillus* spp. [13], and *Actinomyces* spp. [14]). Indeed, due to such antimicrobial potential, along with its low molecular weight, uses and applications of TCN have radically increased in the last 30 years [12]. However, neat TCN incorporation in resin-based materials could lead to a high leachability that can cause a rapid decrease of the antimicrobial properties of such materials. To overcome this issue, TCN was previously incorporated in specific “vehicles” known as nanotubes, in order to achieve a slower and more controlled release of such an antibacterial agent [3,11].

Halloysite nanotubes (HNT) are natural aluminosilicates with a hollow tubular structure [3], which are typically used as a reinforcing nano-filler to improve some mechanical properties of resin-composites [15] such as tensile strength, flexural strength, storage modulus [16], as well as microhardness and bond strength [17]. HNT is a “green” biocompatible nanomaterial characterized by very low cytotoxicity [18]. Furthermore, it acts as “biologically-safe” reservoirs for the encapsulation and controlled release of a variety of therapeutic drugs [15,19], bioactive molecules [20] and matrix metalloproteinase inhibitors [21,22]. Active principles released by halloysite may last 30 to 100 times more than when these were incorporated alone or using some different nanocarriers in polymer nanocomposite [23,24].

Thus, the aim of the present study was to evaluate in vitro the effects of triclosan-encapsulated halloysite nanotubes (HNT/TCN) on the physical-chemical and microbiological properties of an experimental micro-hybrid resin dental composite.

The null hypothesis tested in this study was that the inclusion of HNT-TCN would not influence the physicochemical and microbiological properties of such an experimental resin composite.

## 2. Material and Methods

### 2.1. Material

Halloysite nanotubes (Al_2_Si_2_O_5_(OH)_4_·2H_2_O) with a diameter of 30–70 nm and length of 1–3 μm (Sigma-Aldrich, St. Louis, MO, USA) were treated with a silane solution (5 wt.% of 3-metacryloxypropyltrimetoxysilane and 95 wt.% acetone) at 110 °C for 24 h. Subsequently, these were mixed [1:1 ratio] with 2,4,4-Trichloro-2-hydroxydiphenyl ether (TCN: Triclosan, Fagron, Rotterdam, SH, The Netherlands) under constant shaking for 1 h [3]. The mixture was then dispersed in 95 wt.% pure ethanol (0.03 mg/mL^−1^) and ultasonicated for 1 h. The nanoparticles were finally desiccated for 10 days at 30 °C to ensure complete evaporation of the residual solvents. The HNT/TCN nanoparticles obtained after such processing method were finally prepared and characterized using a Transmission Electron Microscope (TEM) JEM 120 Exll (JEOL, Tokyo, Japan) at 80 kV at a magnification X 300,000.

An experimental resin composite was created by mixing 75 wt.% Bis-GMA (2,2-bis-[4-(hydroxyl-3-methacryloxy-propyloxy)phenyl]propane) and 25 wt.% triethylene glycol dimethacrylate (TEGDMA) (Sigma-Aldrich, St. Louis, MO, USA) under continuous agitation and sonication for 30 min. Camphorquinone (CQ), ethyl4-dimethylaminobenzoate (EDAB), and diphenyliodoniumhexafluorophosphate (DPIHFP; Milwaukee, MI, USA) were also added at 1 mol % to obtain a light-curable resin-based material. Incorporation of 8 wt.% of HNT/TCN and 72 wt.% silica micro-hybrid filler was performed by stirring for 12 h under continuous sonication of the experimental resin composite. 

The control experimental resin composite was formulated with the same organic matrix and silica micro-hybrid filler (80 wt.%), but without the use of the HNT/TCN nanotubes (HNT/TCN-0%) (Table 1).

A commercial nano-filled resin composite was used in this study as a control group. According to the manufacturer (3M ESPE), this material has nanoclusters of zirconia (4–11 nm) and silica (20 nm) nanoparticles, along with micro-filled silica/zirconia particles (0.6 mm) (Table 1).

### 2.2. Degree of Conversion (DC)

Three discs were created for each composite used in this study using teflon molds (6 mm in diameter × 2 mm thick) and light-cured in the absence of oxygen for 40 s under an acetate transparent strip using a light-curing system (1200 mW/cm^2^, Bluephase, Ivoclar Vivadent, Schaan, Liechtenstein), at a standardized 2-mm distance. The degree of conversion (DC) of each material tested in this study was evaluated through micro-Raman spectroscopy (Xplora Horiba, Paris, France) in the range between 1590 and 1670 cm^−1^ using the 638 nm laser emission wavelength, 5 s acquisition time and 10 accumulations [11,25]. Three specimens for each group were analyzed at a standardized room temperature of 23 ± 1 °C. DC was calculated as described in a previous study by Rodrigues et al. [25] on the intensity of the C=C stretching vibrations (peakheight) at 1635 cm^−1^ and using the symmetric ring stretching at 1608 cm^−1^ from the polymerized and non-polymerized specimens.

(1) $$DC\%=\left(1-\left(\frac{R\;cured}{R\;uncured}\right)\right)\;\times \;100$$

### 2.3. Flexural Strength and Flexural Modulus

The flexural strength (FS) and flexural modulus (FM) (*n* = 5) evaluation was performed according to ISO 4049/2000 [26] using a universal mechanical testing machine (Instron 3345, Canton, MA, EUA). Specimens with standard dimensions of 25 × 2 × 2 mm were prepared using a Teflon split mold. A polyester strip and a glass slide covered the resin-composite, and the light tip guide was placed over the center of the mold, to light-cure the specimens for 40 s as described in Section 2.2. After irradiation, the specimens were removed from the molds and carefully polishedusing a 320 grit SiC abrasive paper. All of the specimens were stored in water at 37 °C for 24 h. The specimens were positioned in a 3-point bending apparatus with 2 parallel supports with a distance of 20 mm. The specimens were loaded until fracture with a 500 Kgf load cell at a cross-head speed of 0.05 mm/min. The flexural strength (MPa) was calculated using the following formula:

(2) $$\sigma \;=\;\frac{3L\;\times \;F_{max}}{2w\;\times \;h^{2}}$$

*L* is the distance between the parallel supports (mm); *F_max_* is the load at fracture (N); *w* is the width (mm), and *h* is the height (mm).

The flexural modulus (GPa) was calculated using the following formula:

(3) $$E_{f}\;=\;\frac{{F\;\times \;L}^{3}}{{4wh}^{3}d}$$

*F* and *d* stands for the load and deflection increment, respectively, between 2 specific points in the elastic portion of the curves (N and mm); *L* is the distance between the parallel supports (mm); *w* is the width (mm), *h* is the height (mm).

### 2.4. Dynamic Thermomechanical Analysis (DMA)

Three specimens (8 mm × 2 mm × 2 mm) were prepared for each group (Table 1) and light-cured as previously described. A DMA system (Mettler Toledo, Columbus, OH, USA), equipped with a single bending cantilever was used to determine the mechanical properties in clamped mode. The viscoelastic properties were characterized by applying a sinusoidal deformation force to the material under dynamic conditions: Temperature, time, frequency, stress, or a combination of these parameters. The storage modulus (E′), glass transition temperature (Tg) and tangent delta (TAN-δ) of the tested materials were evaluated at different temperatures under cyclic stress (frequency of 2.0 Hz and amplitude of 10 µm) and from 50 to 800 °C at the heating rate of 2 °C min^−1^. The TAN-δ value represents the damping properties of the material, serving as an indicator of all types of molecular motions and phase transitions. 

### 2.5. Thermogravimetric Analysis (TGA)

A further three specimens were prepared for each group (Table 1) (mass of 10 mg). A thermogravimetric analysis was performed to determine the thermal degradation and the weight percentage of fillers resin-composites. A thermal program from 30 to 193 °C at the heating rate of 2 °C min^−1^ in nitrogen atmosphere determined the weight changes as a function of time and temperature. Thermogravimetric analysis was performed using the Pyris 1 TGA (SDTA851—Mettler Toledo) thermal analyzer.

### 2.6. Polymerization Stress Measurements (PS)

Poly(methyl methacrylate) rods, 5 mm in diameter and 13 or 28 mm in length, had one of their flat surfaces sandblasted with 250 μm alumina. On the shorter rod, to allow for the highest possible light transmission during photoactivation, the opposite surface was polished with silicone SiC papers (600, 1200, and 2000 grit) followed by felt disks with 1μm alumina paste (Alumina 3, ATM, Altenkirchen, Germany). The sandblasted surfaces received a layer of methylmethacrylate (JET Acrilico Auto Polimerizante, Sao Paulo, Brazil), followed by two thin layers of unfilled resin (Scotchbond Multi-Purpose Plus, bottle 3, 3M ESPE). 

The resin composite was light-cured as previously described for 40 s. The rods were attached to the opposing clamps of a universal testing machine (Instron 5565, Canton, MA, USA) with the treated surfaces, facing each other with a 1-mm gap. The resin composite was inserted into the gap and shaped into a cylinder following the perimeter of the rods. An extensometer (0.1 μm resolution), attached to the rods (Instron 2630-101, Bucks, UK) in order to assess the height of the specimen, provided the feedback to the testing machine to keep the height constant. Therefore, the force registered by the load cell was necessary to counteract the polymerization shrinkage to maintain the specimen’s initial height. A hollow stainless steel fixture with a lateral slot attached the short rod to the testing machine, allowing the tip of the light guide to be positioned in contact with the polished surface of the rod. Force development was monitored for 10 min from the beginning of the photoactivation; the nominal stress was calculated by dividing the maximum force value by the cross-section area of the rod. Five specimens were tested for each tested material [27].

### 2.7. Microbiology Assay

*Streptococcus mutans* (*S. mutans*) UA159 (ATTCC) was obtained from single colonies isolated on blood agar plates, inoculated in Tryptone yeast-extract broth containing 1% glucose (*w*/*v*) and incubated for 18 h at 37 °C under micro-aerophilic conditions in partial atmosphere of 5% CO_2_. 

To analyze antimicrobial effects, blocks (4 × 4 × 2 mm) of each group were produced (Table 1). Materials were dispensed in a silicone mold, covered with a polyester tape and then submitted to digital pressure for 2 s to better accommodate the material, with curing light being activated for 40 s. Specimens were sterilized by exposure to Plasma Hydrogen Peroxide before starting biofilm formation. Mono-species *S. mutans* biofilms were formed on blocks placed in bath cultures at 37 °C in 5 % CO_2_ up to 5 days in 24-well polystyrene plates. The biofilms grew in tryptone yeast-extract broth containing 1% sucrose (*w*/*w*) and were kept undisturbed for 24 h to allow an initial biofilm formation. During the biofilm formation period, once daily the discs were dip-washed three times in a plate containing NaCl 0.89% solution to remove the loosely bound biofilm and they were transferred to new 24-well plates with sterile medium. The blocks of each experimental group were removed after 5 days of initial biofilm formation and transferred to pre-weighed microtubes containing 1 mL of NaCl 0.89 % solution. Biofilms were then dispersed with 3 pulses of 15 s with 15 s of interval at a 7-W output (Branson Sonifier 150; Branson Ultrasonics, Danbury, CT, USA). An aliquot (0.05 mL) of the homogenized biofilm was serially diluted (10^−1^–10^−7^) and plated onto blood agar plates. Plates were then incubated at 37 °C, 5% CO_2_ for 48 h, before enumerating viable microorganisms. Results were expressed as colony forming units (CFU)/mL and transformed in log_10_ CFU to reduce variance heterogeneity [28].

To determine the biofilm dry weight, 200 μL aliquots of the initial biofilm suspension were transferred to pre-weighed tubes and dehydrated with ethanol solutions (99 %). The tubes were centrifuged, and the supernatants were discarded before the pellet was dried into a desiccator (P_2_O_5_) for 24 h and weighted (±0.00001 mg). The dry weight of the biofilm was determined by calculating the weight in the tube (initial weight − final weight) and in the original suspension (dry weight in 1 mL = dry weight in 200 μL × 5) [29]. 

### 2.8. Statistical Analysis

Physicochemical properties data was submitted to analysis of variance with one factor (One way-ANOVA), followed by Tukey test. For analyzing antimicrobial effects was performed analysis of variance with one factor (One way-ANOVA). Significance level was set at 5%. The program used to perform the analyses was IBM SPSS Statistics Version 20.0 (Armonk, NY, USA).

## 3. Results

**Figure 1:**
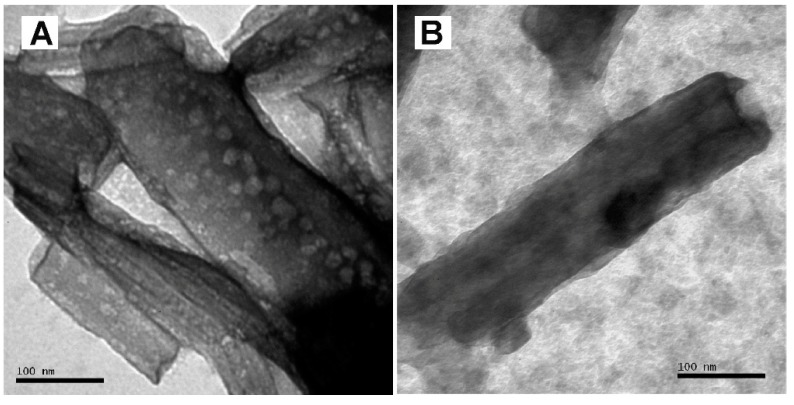
(**A**) TEM image that shows the presence of TCN nanoparticles with diameter of 5–10 nm. (**B**) TEM image of a nanotube with its inner-surface of 40–50 nm diameter range and outer-surface of 90 nm diameter.

**Figure 2:**
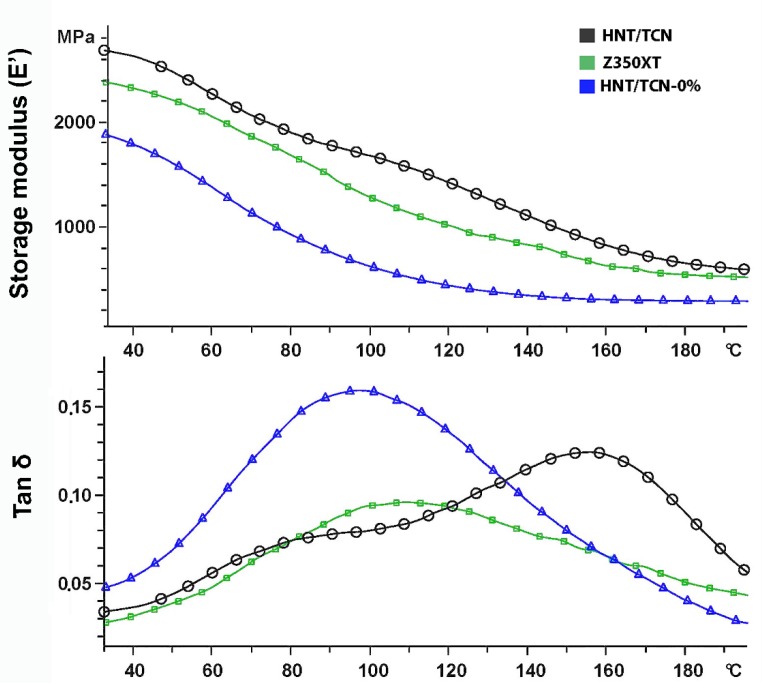
DMA curves of the specimens showed that the MPa, TAN δ at Tg for HNT/TCN resin composite presented a lower TAN δ at Tg when compared to the control composite containing no HNT/TCN-0%. The glass transition temperature (Tg) of HNT/TCN is higher than the other resin composite.

**Figure 3:**
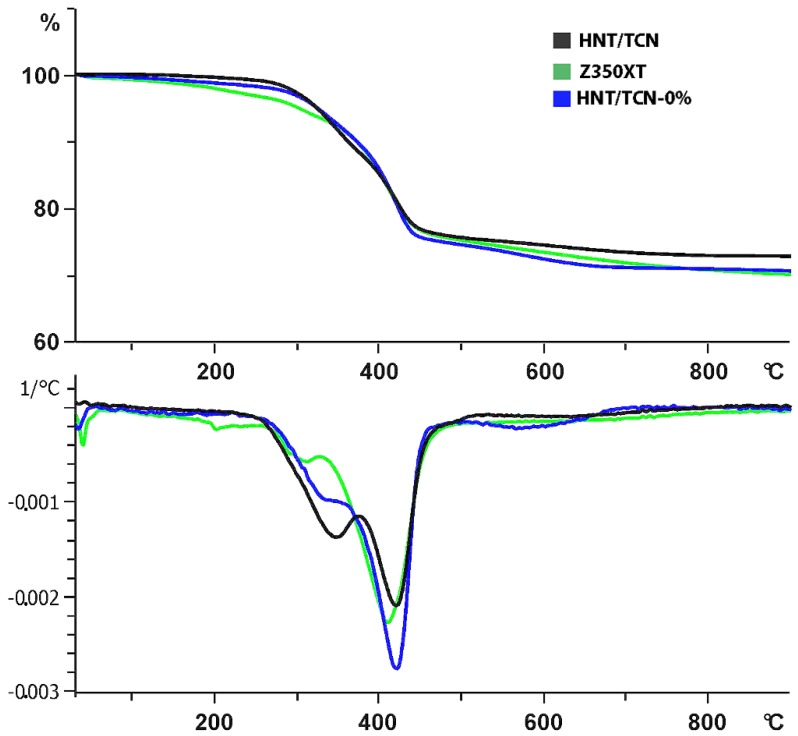
TGA curves of the specimens. Curve of the weight loss (%) showing that HNT/TCN resin composite presented a lower weight loss than other groups. The second curve below shows that there were two steps of degradation for each group.

TEM analysis showed that TCN was successfully deposited inside the lumen of the HNTs (Figure 1A,B). Means and standard deviations of physicochemical properties of the tested materials are presented in Table 2 and Table 3. To summarize, the degree of conversion (DC) test showed that there was no significant difference between the tested materials (*p* = 0.879). The flexural strength of HNT/TCN and that of Z350XT was greater than attained with the control HNT/TCN-0% (*p* = 0.005) composite. However, no significant difference was encountered between the storage modulus (*E_f_*) of the three tested groups (Figure 2). The maximum polymerization stress obtained in the specimens created with HNT/TCN composite was significantly (*p* < 0.05) greater than that observed in the control HNT/TCN-free resin composite. The DMA assessment showed no significant differences between the three tested composites for the TAN δ at Tg (*p* = 0.006) and Tg (*p* = 0) (Table 3). The TGA curves (Figure 3) obtained in nitrogen atmosphere showed that there was no significant difference between the three tested composites on the first degradation step (*p* > 0.05): HNT/TCN (296 °C), HNT/TCN-0% (301 °C) and the commercial resin composite (286 °C). Moreover, on the second degradation step, no significant difference was observed: HNT/TCN (419 °C), HNT/TCN-0% (426 °C) and the commercial resin composite (415 °C). There was no difference (*p* = 0.317) between the weight loss of the composites HNT/TCN (25.7%), HNT/TCN (24.5%) and the commercial resin composite (30%) (Table 3).

The results of the microbiological test are presented in Table 4. There were no statistically significant differences (*p* = 0.977) between the experimental groups for CFUs and (*p* = 0.557) for dry weight.

## 4. Discussion

Recent studies have demonstrated that the incorporation of HNTs (5 wt.%) in resin-based materials (i.e., dental adhesive systems and enamel infiltrants) could improve their micro-hardness, flexural strength [15] and maximum polymerization rate [11]. However, if the concentration of HNTs is higher than 10 wt.% it is likely to attain a decrease of both flexural strength [15] and maximum polymerization rate [11]. The reason of such outcomes has been attributed to the behavior of such nanotubes to agglomerate in micro-cluster; this causes interference in the mechanism of interaction between nanotubes and the polymer matrix [30]. The null hypothesis that the inclusion of HNT-TCN at concentration of 8% into an experimental resin composite would have increased the physicochemical and microbiological properties must be partially accepted since some physicochemical properties were improved, although no significant differences were attained in terms of antibacterial activity (CFUs) and dry weight after 5 days of initial biofilm formation.

The physicochemical results obtained in this study are in agreement with those recently presented by Degrazia et al. [11]. Indeed, the organic matrix structure and the characteristic of fillers employed in the formulation of composite materials exert a direct influence on the surface roughness, degree of conversion, finishing, and polishing procedures; this may influence the surface quality of resin composite when applied in clinical scenarios [31]. Moreover, it is believed that if a higher degree of conversion is achieved, it is possible to extend the long-term stability and longevity of resin composites, especially in those cases when relatively “short” light-curing periods are performed [32]. However, despite the absence of statistical significance, the HNT/TCN composite presented greater numerical degree of conversion compared to the two control composites tested in this study. It is important to consider that for monomers with the same functionality, the higher the conversion of double bond monomers, the greater the mechanical strength of the cured resin [33]. Indeed, it has been already demonstrated that during the light-curing process HNT/TCN nanotubes may increase the intermolecular interactions between monomers during polymerization [30], due to the C=O and Al-O-H groups present on the inner and outer surface of the HNTs [34]. In general, in case of greater cross-link conversion degree, the conversion of monomers may advance and increase the density of the polymer network [35].

The dynamic mechanical analysis is usually employed for the evaluation of the glass transition temperature (Tg) of resin-based materials, since this allows a more “in-depth” knowledge of the network homogeneity and cross-linking density [36]. As previously stated, the addition of nanotubes in resin-based materials may cause the formation of intermolecular interactions (hydrogen bond formation between hydroxyl groups) between the outer surface of HNTs and Bis-GMA molecule by hydroxyl groups [30].

It has been reported that, low values of TAN δ at specific Tg values indicate a better interfacial adhesion between the organic matrix and the filler [35]. In the present study, the glass transition temperature of the tested materials showed that the HNT/TCN composite presented a lower TAN δ at Tg when compared to the control composite containing no HNT. This outcome indicates that the nanotubes may enhance the chemical interaction during polymerization reaction between the organic matrix and the inorganic fillers (Figure 2 and Figure 3).

Furthermore, the strength, modulus and impact resistance of resin-based materials can be increased when these are doped with HNTs, even at relative low concentration (5 wt.%) [23]. According to the ISO 4049/2000 standard [26], the flexural strengths of universal resin-based restorative materials should be higher than 80 MPa. The Flexural strength test (Table 2) showed that FS of the commercial nanofilled composite and that of the experimental composite HNT/TCN were statistically higher than FS obtained with the control composite HNT/TCN-0%; the latter did not meet the minimal required values of ISO 4049/2000. High flexural strength is necessary to prevent cohesive fractures within the bulk material, especially when considering posterior restorations [37,38]. However, HNT/TCN presented FS values similar to that of the modern commercial composite used in this study. However, although the similarity in flexural strength, the elastic modulus of the Filtek Z350 XT attained in our study was lower than that reported by Rosa et al., 2012 [39]. The main reason for such a difference can be attributed to the different light-curing protocol used in our study.

A further important characteristic that may influence the stress development is the flexural modulus (FM); this is often associated with the composition of the tested material. In this study, the FM tests showed no significant difference between the tested groups (Table 2). Correlation between the FM and the polymerization stress values is a valid simplified approach [38]. However, the limitations of this in vitro test are related to the fact that the light needed to pass through the acrylic rod, as well as the 1 mm thick material before reaching the upper rod in order to create the bonding between the materials and the rods [40]. Indeed, in this study the high standard deviation of polymerization stress test obtained in the HNT/TCN resin composite may be related to the compliance of the test. Tensilometer test configurations can also present some limitations when considering a clinical scenario and the configuration of the cavities [41]. Nonetheless, it was previously reported that it is a valid method to simulate the conditions present in small cavities, where the volume of the resin composite is restricted; this may represent the clinical situation in minimally invasive restorative procedures [42].

Resin composites with lower values of the polymerization stress are those having the lowest elastic modulus; this is probably due to the greater deformation capacity of these latter materials [43]. The addition of HNT/TCN in the experimental resin composite resulted in a significant increase in PS (Table 2). It is known that resin-composites with high modulus of elasticity may generate stiffer restorations; such conditions may increase the effect of the polymerization stress on the tooth-composite interfaces [44]. Conversely, resin composites with a lower elastic modulus create less stress on the interface, although these may lack full dimensional recovery to withstand the masticatory load [44]. Indeed, it can be observed that despite the higher polymerization stress value of HNT/TCN, FM values had the tendency to be higher than the other two materials tested in this study. Another reason HNT/TCN composite showed higher polymerization stress may be associated with the lower micro-hybrid silica filler content (72%) compared to the HNT-free control composite, which had 80 wt.% of micro-hybrid filler.

To test the antibacterial effect, a test was carried out with 5 days of biofilm growth. Degrazia and collaborators [11], showed that their resin-based material doped with HNT/TCN had antibacterial effect up to 72h; antibacterial properties can be also evaluated after 24 h of biofilm growth. It is suggested that the antibacterial effect can be obtained by direct contact of *S. mutans* with the inhibitory agent present in the resin composite (TCN in case of the present work). In agreement with this idea, Feitosa and collaborators [22] demonstrated strong antibacterial activity when *S. mutans* was in direct contact with doxycycline-encapsulated nanotube-modified dentin adhesive after 24 h. In the present study, no statistically significant difference was found between the CFUs values of the 3 tested composites biofilm formed over a 5-day period (*p* = 0.977) (Table 4). It seems that the decrease in the inhibitory antibacterial effect overtime might be related to the inability of such agent from the resin composite to reach the whole thickness of the plaque, in particular the outer layers of bacteria. Probably, the absence in degradation and wearing of the material seems to be considered as an advantage. Due to the interestingly low CFU of the HNT/TCN, in the present work, we believe that the daily removal of the biofilm can reduce its thickness and allow the direct contact of the agent with the bacteria and promoting a more effective antibacterial action. Furthermore, it is possible that current antibacterial tests performed in a laboratory may not be totally appropriate for the evaluation of resin composites, unless accompanied with aging protocols that allow the release of therapeutic agents such as the TCN.

Future studies are suggested to improve the development of antimicrobial materials and the understanding of the relationship between their formulations, morphology and properties, to promote the longevity (shelf-life and ageing) of resin-based materials restorations. Moreover, studies including its rheological properties with alternative methods for synthesis and nanotubes can extend the range of application of such materials as well the satisfaction of as patients and clinicians.

## 5. Conclusions

It can be concluded that:

- Incorporation of 8 wt.% seems to be a satisfactory formulation of halloysite nanotube for achieving appropriate mechanical properties for an experimental resin micro-hybrid composite without affecting the viscosity and the material and increase the risk for phase separation;
- The experimental resin composite containing 8 wt.% halloysite nanotube doped with triclosan, from a physicochemical point of view, seems to be a suitable restorative material such as the current commercial nano-filled resin-composite;
- Incorporation of triclosan seems to be test-dependent, since it showed no response in mature biofilms as used in this study.

## Figures and Tables

**Table 1:** Composition of the resin composite used in this study.

Name of the Composite	Type of Composite	Manufacturer/Lot No.	Composition
**HNT/TCN-0%**	Experimental resin-composite	-----	**Organic matrix:** Bis-GMA, TEGDMA.
			**Filler type:** Silica micro-hybrid filler 80 wt.%
			**Filler content:** 80 wt.%
**HNT/TCN**	Experimental resin-composite	-----	**Organic matrix:** Bis-GMA, TEGDMA.
			**Filler type:** Silica micro-hydrid filler 72 wt.% and halloysite nanotubes 8 wt.%.
			**Filler content:** 80 wt.%
**Filtek Z-350XT** **(Shade A1D)**	Commercial nano-filled composite	3M ESPE (St Paul, MN, USA)/N702257	**Organic matrix:** Bis-GMA, Bis-EMA, UDMA, TEGDMA
			**Filler type:** Silica and zirconia nanofillers, agglomerated zirconia-silica nanoclusters
			**Filler content:** 82 wt.%

**Table 2:** Results Degree of Conversion (DC), Flexural Modulus (E) and Flexural Strength (FS), Maximum polymerization stress (PS).

Composite	DC (%)	E (GPa)	FS (MPa)	PS (MPa)
**HNT/TCN-0%**	75.9 (5.4)^ A^	6.8 (0.9)^ A^	75.9 (10.1) ^B^	3.6 (0.3) ^B^
**HNT/TCN**	78.5 (2.2)^ A^	7.5 (0.2)^ A^	107.2 (6.6) ^A^	5.4 (0.9) ^A^
**Z350XT**	72.5 (10.6)^ A^	6.8 (0.4)^ A^	101.4 (18.4) ^A^	3.6 (0.3) ^B^

* Different capital letters in column indicate statistical difference (*p *< 0.05).

**Table 3:** Results Glass Transition Temperature (Tg), TAN-δ and Thermogravimetric analysis (TGA).

Composite	Tg (°C)	Tanδ (×10^3^) at Tg	TGA Weight Loss (%)	TGA Temperature of the First Degradation Step (°C)	TGA Temperature of the Second Degradation Step (°C)
**HNT/TCN-0%**	102 (6.56) ^B^	156.7 (0.15) ^A^	27.3 (0.58) ^A^	301 (1.73) ^A^	426 (3.6) ^A^
**HNT/TCN**	154 (4.36) ^A^	106.7 (0.11) ^B^	26.3 (0.58) ^A^	296 (1.0) ^B^	419 (1.15) ^A,B^
**Z350XT**	105.3 (3.51) ^B^	103.3 (0.15) ^B^	27 (1.0) ^A^	286 (2.64) ^C^	415 (5.72) ^B^

* Different capital letters in column indicate statistical difference (*p* < 0.05).

**Table 4:** Results microbiological tests, as colony forming units after 5 days (CFU)/mL/mm^2^ and biofilm dry weight. This test showed no statistical difference between experimental groups and commercial resin composite (*p =* 0.977).

Composite	(CFU)/mL/mm^2^	Dry Weight (g)
HNT/TCN-0%	6.9267 (0.35) ^A^	0.0004 (0.00025) ^A^
HNT/TCN	6.8733 (0.28) ^A^	0.0008 (0.00082) ^A^
Z350XT	6.8867 (0.28) ^A^	0.0003 (0.00006) ^A^

* The same capital letters indicate absence of statistical difference (*p* < 0.05).
